# Concomitant driver mutations in advanced *EGFR*-mutated non-small-cell lung cancer and their impact on erlotinib treatment

**DOI:** 10.18632/oncotarget.25490

**Published:** 2018-05-25

**Authors:** Jan Nyrop Jakobsen, Eric Santoni-Rugiu, Morten Grauslund, Linea Melchior, Jens Benn Sørensen

**Affiliations:** ^1^ Department of Oncology, Copenhagen University Hospital/Rigshospitalet, Copenhagen, Denmark; ^2^ Department of Pathology, Copenhagen University Hospital/Rigshospitalet, Copenhagen, Denmark

**Keywords:** NSCLC, EGFR, erlotinib, tarceva, mutation

## Abstract

**Background:**

Patients with *EGFR*-mutated non-small-cell lung cancer benefit from EGFR tyrosine kinase inhibitors (TKIs) like erlotinib. However, the efficacy may be impaired by driver mutations in other genes.

**Methods:**

Five hundred and fourteen consecutive patients with NSCLC of all stages were tested for *EGFR*-mutations by cobas® EGFR Mutation Test. Fluorescent *in situ* hybridization (FISH) for *MET*-amplification, immunohistochemistry (IHC) for MET- and ALK-expression, and Next Generation Sequencing (NGS) for concomitant driver mutations were performed on *EGFR*-mutated tumor samples from erlotinib-treated patients.

**Results:**

Thirty-six patients (7%) had *EGFR*-mutations, including 2 with intrinsic resistance mutation p.T790M together with the p.L858R sensitizing mutation and 1 harboring the p.G719C/S768I double-mutation. Twenty-three patients had either locally advanced or advanced disease and received first-line erlotinib-treatment. Concomitant driver mutations were found in 15/21 (71%) of NGS-analyzed TKI-treated NSCLCs, involving in 67% of cases *TP53*, in 13% *CTNNB1*, and in 7% *KRAS*, *MET*, *SMAD4*, *PIK3CA*, *FGFR1*, *FGFR3*, *NRAS, DDR2*, and *ERBB4*. No ALK-expression was found, whereas MET-overexpression and *MET*-amplification were observed in 5 and 4 patients, respectively. Objective responses occurred in 17/23 patients (74%), 4 did not respond (17%), and 2 harboring a *SMAD4*-mutation (p.R135^*^(stop)) and a *FGFR3*-mutation (p.D785fs^*^31), respectively, displayed mixed response with simultaneously progressing and responding tumors (8.7%). Thus, *EGFR*-mutated tumors harboring co-mutations were not less likely to respond.

**Conclusion:**

Co-mutations in other cancer-driver genes (oncogenes or tumor suppressor genes) were frequent in *EGFR*-mutated NSCLCs and few cases harbored concomitant activating and resistance *EGFR*-mutations before TKI-treatment. Most co-mutations did not impact the response to first-line erlotinib-treatment, but may represent potential additional therapeutic targets.

## INTRODUCTION

EGFR is a member of the ErbB family of transmembrane receptor tyrosine kinases (TKs) and upon ligand-binding and dimerization undergoes auto-phosphorylation of its intracellular domain, resulting in recruitment of different adaptors and signal-transducers and activation of downstream signaling-pathways. These especially include the RAS-RAF-MEK-MAPK, the PI3K-AKT-PTEN-mTOR, and the STAT pathways, ultimately resulting in cancer-promoting effects, such as increased cell proliferation, survival, protein synthesis, migration, and angiogenesis [1]. Common gain-of-function mutations of *EGFR*, such as most of microdeletions in exon 19 and the point-substitution p.L858R in exon 21, which together represent almost 90% of all *EGFR*-mutations, result in constitutive ligand-independent EGFR-TK activity and oncogenicity as well as increased affinity and sensitivity to the first- and second-generation EGFR-TK inhibitors (TKIs) gefitinib, erlotinib and afatinib. These drugs are currently the standard of care first-line treatment for patients with advanced *EGFR*-mutated NSCLC, with higher response rate (RR) observed for patients with exon 19-microdeletions [1, 2]. Despite initial response to first-line EGFR-TKIs, most patients inevitably become resistant with median progression-free survival (PFS) of 10-12 months. In up to 60% of cases, this is due to the emergence of the secondary p.T790M EGFR-mutation in exon 20, which also is an activating mutation, but possesses increased affinity for ATP, thereby competitively impeding the binding of reversible EGFR-TKIs to the EGFR ATP-binding pocket [3, 4]. The third-generation EGFR-TKI osimertinib is currently the standard of care for treating advanced EGFR-mutant NSCLC with p.T790M-positive acquired TKI-resistance.

Most of the activating *EGFR*-mutations occurring in non-small-cell lung cancer (NSCLC) before treatment are mutually exclusive with those in other oncogenic drivers. However, additional distinct driver alterations such as *ALK*-rearrangement, *KRAS*-mutations, *PIK3CA*-mutations, *MET*-amplification and others were recently reported to co-exist with *EGFR*-mutations in a small percentage of TKI therapy-naïve pulmonary adenocarcinomas (ADCs) [5–13]. A recent database study including 17664 lung cancer patients identified 2-3 concomitant driver mutations in almost 1% of these cases [6].

Importantly, the occurrence of co-mutations in *EGFR* itself or other cancer-drivers at diagnosis may potentially impair the efficacy of tyrosine-kinase-inhibitors (TKIs) and partly explain why approximately 10% of TKI-treated NSCLCs are intrinsically resistant [4].

Consequently, the evaluation of *EGFR*-mutations and *ALK*-rearrangements for selecting NSCLC patients treatable with first-line TKIs may not be sufficient to predict the response to these treatments. Thus, we examined the frequency of an extended panel of cancer-relevant mutations that could potentially reduce the initial response to TKIs in a cohort of newly diagnosed, *EGFR*-mutated, advanced NSCLC of primarily ADC subtype. Additionally, we evaluated the response to erlotinib of *EGFR*-mutated tumors with or without co-mutations.

## RESULTS

### EGFR-mutations and patients characteristics

**Figure 1:**
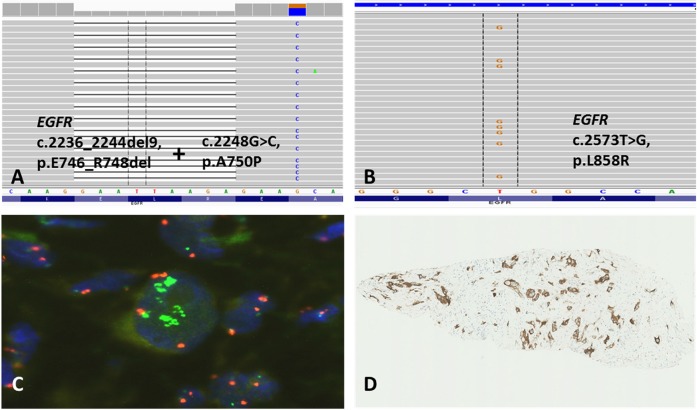
Unusual double p.E746_R748del/p.A750P *EGFR*-mutation identified by NGS in one NSCLC-case (**A**; patient no. 9 in Table 2). Biopsy from NSCLC of ADC subtype with activating p.L858R *EGFR*-mutation (**B**; patient no. 5 in Table 2) identified by NGS, high-level *MET*-amplification detected by FISH (**C**; green, x100), and overexpression of MET receptor assessed by IHC (**D**; x10).

Thirty-six (7%) of the 514 tested NSCLCs displayed *EGFR*-mutations, including two tumors with co-existing p.L858R and p.T790M mutations, one of which was from an operable patient. Of the 514 patients, 283 (55.1%) had advanced stage or were not amenable for definitive local treatment because of poor pulmonary function. Twenty-three of these patients (8.1%) had tumors harboring activating *EGFR*-mutations and were treated with erlotinib, their individual characteristics are shown in Table 1 and histological subtype and type of *EGFR*-mutations in Table 2. Eighteen (78%) of the patients with *EGFR*-mutated tumors were females and five (22%) males, whereas in terms of smoking habit, 18 (78%) were never/previous smokers and 5 (22%) current smokers. According to established criteria [14], the specimens from 20 of the *EGFR*-mutant cases (87%) were diagnosed as lung ADCs, based on the presence of acinar/papillary/micropapillary/solid epithelial structures, mucin production, and immunohistochemical markers (all were CK7+/TTF1+/CK5-/p40-), whereas one specimen (4%) was classified as SCC because of lack of mucin production and marker status (CK7-/TTF1-/CK5+/p40+). The specimens of the other two *EGFR*-mutant cases (9%) were classified on biopsies as most likely adenosquamous carcinomas (ADSC), based on the co-existence of cells with mucin production, keratinization and ADC/SCC immunohistochemical markers (CK7+/TTF1+/CK5+/p40+). Seven patients had a single p.L858R mutation, 14 an exon 19-microdeletion, one harbored the p.G719C/p.S768I double mutation, one p.L858R combined with the intrinsic erlotinib-resistant p.T790M mutation, and one the unusual p.E746_R748del/p.A750P combination (Figure 1A). Taken together, these results are consistent with the well-established notions that, regardless of ethnicity, the frequency of *EGFR*-mutations is higher among women, never/light smokers, and with NSCLC of ADC type, and that the exon 19-microdeletions and the p.L858R substitution in exon 21 are the most frequent *EGFR*-mutations in NSCLC [1].

**Table 1:** Individual characteristics and outcome of 23 *EGFR*-mutation positive, treatment naïve NSCLC patients treated with erlotinib

Patient no.	Gender	Age	PS	Smoking status	Stage	Objective response to erlotinib	PFS (months)	OS (months)
1	Female	75	1	Never	IIIA	PR	24	24
2	Female	58	1	Current	IV	PR	19	41
3	Female	61	0	Never	IV	PR	5	7
4	Female	62	0	Previous	IV	PR	8	25
5	Female	76	1	Current	IV	PR	7	16
6	Female	81	0	Previous	IIIA	PR	20	50 (Alive)
7	Male	83	1	Never	IV	PR	18	29
8	Female	62	1	Never	IV	NC	10	47
9	Female	59	0	Previous	IIIB	PR	17	34
10	Female	50	0	Current	IV	NC	20	35
11	Female	83	1	Previous	IV	PR	17	32
12	Female	60	0	Never	IV	Mixed	15	21
13	Female	53	0	Never	IV	NC	14	23
14	Female	56	0	Current	IV	PR	8	28
15	Female	47	1	Previous	IV	PR	6	9
16	Female	82	2	Never	IV	PR	8	15
17	Female	81	1	Never	IIIA	PR	9	22
18	Male	67	1	Previous	IV	PR	8	17
19	Male	63	0	Previous	IV	NC	9	23
20	Female	63	0	Never	IV	CR	13	33
21	Female	59	1	Previous	IV	CR	8	19
22	Male	75	1	Previous	IIIB	PR	8	16
23	Male	49	1	Current	IV	Mixed	1	10

PS: WHO Performance status; CR: Complete response; PR: Partial response; NC: No change.

**Table 2:** Histological subtype and biomarker status in 23 *EGFR*- mutation- positive NSCLC patients treated with erlotinib

Patient no.	NSCLC histotype	*EGFR*-mutations^ (c.: cDNA sequence; p.: protein sequence)	*MET*-amplification^§^	MET protein overexpression^§§^	*TP53* mutation	Other mutations
1	ADSC	c.2573T>G, p.L858R			c.473G>T, p.R158L (pathogenic)	*KRAS*: c.35G>T, p.G12V (pathogenic)
2	ADC	c.2155G>T, p.G719C + c.2303G>T, p.S768I	Intermediate-level of amplification	3+ in 80% of tumor cells	c.503A>G, p.H168R (likely-pathogenic)	
3	ADC	c.2573T>G, p.L858R				
4	ADSC	c.2573T>G, p.L858R	High-level amplification	3+ in 100% of tumor cells		
5	ADC	c.2573T>G, p.L858R	High-level amplification	3+ in 70% of tumor cells		
6	ADC	c.2239_2253del15, p.L747_T751delLREAT	*Not enough cells for analysis*	*Not enough cells for analysis*		
7	ADC	c.2239_2253del15, p.L747_T751delLREAT			c.723Cdel, p.S241fs^*^ (unknown)	
8	ADC	c.2573T>G, p.L858R + c.2369C>T, p.T790M				
9	ADC	c.2236_2244del9, p.E746_R748del + c.2248G>C, p.A750P				*MET*: c.3029C>T, p.T1010I (pathogenic)
10	ADC	c.2213-2230dup, p.I744_K745insKIPVAI	Low-level amplification	3+ in 100% of tumor cells	c.463A>C, p.T155P (neutral)	
11	SCC	c.2235_2249del15, p.E746_A750delELREA				*Not sufficient DNA for NGS analysis*
12	ADC	c.2573T>G, p.L858R				*SMAD4*: c.403C>T, p.R135^*^ (stop) (pathogenic)
13	ADC	Exon 19 del (by Cobas)				*Not sufficient DNA for NGS analysis*
14	ADC	c.2236_2250del15insTTA, p.E746-T750delinsL			c.734G>A, p.G245D (pathogenic)	
15	ADC	c.2235_2249del15, p.E746_A750delELREA			c.818G>T, p.R273L (pathogenic)	*NRAS*: c.401C>T, p.A134V (unknown); *DDR2*: c.1549G>C, p.G517R (unknown); *ERBB4*: c.716C>T, p.S239P (pathogenic)
16	ADC	c.2573T>G, p.L858R			c.216Cdel, p.P74fs^*^ (unknown)	*PIK3CA*: c.1633G>A, p.E545K (pathogenic)
17	ADC	c.2240_2257del18, p.L747_P753delinsS				*CTNNB1*: c.98C>G, p.S33C (pathogenic)
18	ADC	c.2235_2249del15, p.E746_A750delELREA		3+ in 100% of tumor cells	c.535C>T, p.H179Y (pathogenic)	
19	ADC	c.2236_2250del15, p.E746_A750delELREA			c.403T>G, p.C135G (pathogenic)	
20	ADC	c.2235_2249del15, p.E746_A750delELREA			c.376-1G>T (Substitution - intronic) (pathogenic)	*FGFR1*: c.373_374insTCA, p.S125-E126insS (unknown)
21	ADC	c.2235_2249del15, p.E746_A750delELREA				
22	ADC	c.2573T>G, p.L858R				*CTNNB1*: c.110C>T, p.S37F (pathogenic)
23	ADC	c.2235_2249del15, p.E746_A750delELREA	.			*FGFR3*: c.2349_2350delAG, p.D785fs^*^31 (unknown)

ADC: Adenocarcinoma; SCC: Squamous cell carcinoma; ADSC: Adenosquamous carcinoma

In parenthesis the pathogenic prediction of the mutations according to the catalogue of somatic mutations in cancer (COSMIC) at http://cancer.sanger.ac.uk/cosmic. ^All identified *EGFR*-mutations were COSMIC-verified as being pathogenic.

§ For clarity only cases with some level of *MET*-amplification (high-, intermediate-, low-level) are shown; empty rows = no *MET*-amplification, as defined in Materials and methods.

§§ For clarity only cases with MET-overexpression (3+) and % of overexpressing cells are shown, empty rows = cases with MET-expression 2+, 1+ or 0, as defined in Materials and methods.

### NGS findings

DNA for NGS was available from 21 of the 23 *EGFR*-mutated cases (Table 2). Complete concordance was observed between *EGFR*-mutation-status by Cobas® EGFR Mutation Test and NGS. However, one sample identified by Cobas®Test as exon 19-microdeletion turned out to be an exon 19-insertion resulting in 6-amino-acid duplication (c.2213-2230dup, p.I744_K745insKIPVAI) by NGS analysis. Fifteen of the 21 NGS-analyzed cases displayed co-mutations in other cancer-relevant genes (Table 2), with *TP53*-mutations being the most represented (n=10, 67%, subdivided in 7 missense mutations, 1 intronic substitution, 2 previously unreported frameshift deletions). Other mutations were identified in *CTNNB1* (n=2, 13%), *KRAS*, *FGFR1*, *FGFR3*, *MET*, *SMAD4*, *PIK3CA*, *NRAS*, *DDR2*, *ERBB4* (all n=1, 7%), with the latter 3 gene-mutations concomitantly present in the same tumor (patient 15, Table 2). The spectrum and pathogenic prediction of these co-mutations according to the catalogue of somatic mutations in cancer (COSMIC, http://cancer.sanger.ac.uk/cosmic) are indicated in Table 2.

### FISH and IHC

None of the 23 *EGFR*-mutated cases showed expression of ALK-fusion protein. FISH and IHC for *MET* were feasible in 22 of the cases. We detected significant variation concerning average *MET* GCN per tumor cell nucleus (range 1.62-13.74; mean 3.81) and *MET*/CEN7 ratio (range 0.74-4.58; mean 1.31), which is consistent with other recent results in treatment-naïve NSCLCs [11].

Two of the FISH-analyzed cases (9.1%) exhibited high-level *MET* gene-amplification concomitant with the p.L858R *EGFR*-mutation (Figure 1B-1D), while another case (4.5%) showed intermediate-level of *MET* GCN gain together with the above-mentioned p.G719C/p.S768I double mutation. Additionally, the sample (4.5%) harboring the above-mentioned *EGFR* exon 19-insertion p.I744_K745insKIPVAI had low-level of *MET* GCN gain (Table 2). The remaining 18 cases (81.8%) were negative for *MET*-amplification. The 4 cases with *MET*-amplification, regardless of the GCN increase level, showed MET-overexpression by IHC (Table 2, Figure 1D). In addition, one *EGFR*-mutated tumor exhibited MET-overexpression without *MET*-amplification (Table 2), suggesting other mechanisms increasing the receptor expression in this case.

### Clinical outcomes

**Figure 2:**
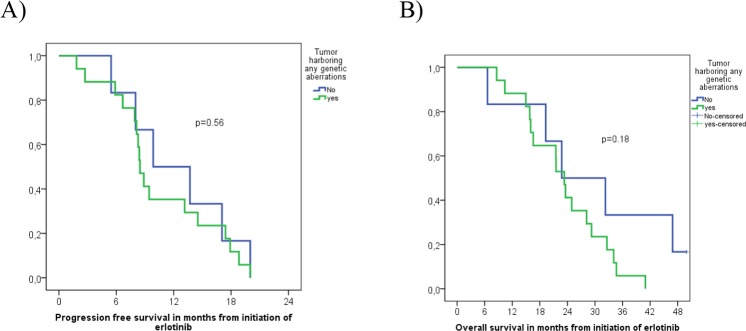
Progression free survival **(A)** and Overall survival **(B)** of 23 *EGFR*-mutation positive NSCLC patients with or without identified concomitant genetic aberrations.

Among the 23 *EGFR*-mutated patients treated with erlotinib, 17 (74%) responded, 4 (17%) had no OR, and 2 (9%) harboring a *SMAD4*-mutation (p.R135^*^(stop)) and a *FGFR3*-mutation (p.D785fs^*^31), respectively, showed mixed response with simultaneously progressing and responding tumor lesions (Tables 1 and 2). The above-named tumor harboring the double p.L858R/p.T790M *EGFR*-mutation, did not respond to erlotinib, while another case with p.L858R did, despite concurrently harboring the p.G12V *KRAS*-mutation and p.R158L *TP53*-mutation. Median PFS was 8.8 months, 95%CI [7.3; 10.5] and OS was 23.3 months, 95%CI [20.1; 26.6] among all 23 patients harboring *EGFR*-mutations. No significant differences in PFS (8.5 months, 95%CI [7.7; 9.3] vs. 9.9 months, 95%CI [3.0; 16.7]) (p=0.56) or OS (23.3 months, 95%CI [20.5; 26.2] vs. 22.8 months, 95%CI [7.2; 38.3]) (p=0.18) were observed when comparing *EGFR-mutated* tumors with or without concomitant genetic aberrations (Figure 2). However, 2 tumors with insufficient DNA for NGS and 1 tumor with too few cells for FISH and IHC were included in the analysis as being without concomitant mutations.

## DISCUSSION

Certain *EGFR*-mutated NSCLCs are inherently resistant to TKI-inhibitors and NSCLCs initially responding will ultimately become TKI-resistant and progress. In our study, 7% (36/514) of tested NSCLCs of all stages and 8% (23/283) of NSCLCs in advanced stage harbored *EGFR*-mutations, consistent with previous data concerning Danish patients [15].

Among the advanced erlotinib-treated NSCLCs, one simultaneously carried the p.L858R and the intrinsic erlotinib-resistant p.T790M *EGFR*-mutations, but no mutations in other analyzed genes. Known as the most common mechanism of acquired resistance to first-/second-generation EGFR-TKIs, p.T790M occurrence at baseline seems frequently associated with lack of OR and poor outcome in patients treated with these TKIs [3, 4]. Accordingly, our p.L858R/p.T790M case displayed no OR. Another erlotinib-treated case harbored the double p.G719C/p.S768I *EGFR*-mutation, each considered uncommon and reportedly less sensitive to gefitinib/erlotinib than more common *EGFR*-mutations [16, 17]. Yet, p.G719C and p.S768I may coexist more frequently than other rare mutations in NSCLC [16, 18]. Moreover, patients with *EGFR* co-mutations exhibit shorter PFS and lower RR than patients with single *EGFR*-mutations [19]. Although S768I alone or concomitant with exon 18 G719X does not necessarily appear to be sensitive to erlotinib [16, 17] it was shown to respond to afatinib [20, 21]. Our p.G719C/p.S768I case also showed intermediate-level of *MET*-amplification, MET-overexpression, and the fairly rare p.H168R *TP53*-mutation, nevertheless it did display an OR to erlotinib. Thus, given the rarity and variable response of TKI-treated cases with exon 20 S768I, the exact prognostic and predictive role of this mutation in *EGFR*-mutant NSCLC seems still unclear. A separate *EGFR*-mutant NSCLC carried the unusual combination of the two rare mutations p.E746_R748del and p.A750P in *EGFR* exon 19, together with the p.T1010I point-mutation in the *MET*-gene. The sensitivity of these two exon 19-mutations to EGFR-TKIs is poorly described, while the *MET*-substitution has been reported to be associated with compromised response to these drugs [22]. In any case, this patient did show a partial response to erlotinib with PFS longer than 17 months. Another case displayed an *EGFR* exon 19-insertion resulting in 6 amino acid duplication (p.I744_K745insKIPVAI) together with a missense *TP53*-mutation and low-level *MET*-amplification. *EGFR* exon 19-insertions have been reported in only 0.26% and 0.11% of large Caucasian and Asian NSCLC-cohorts, respectively [23, 24], thus their response to EGFR-TKIs is uncertain. A recent meta-analysis of the few reported patients with these mutations suggested that they had lower RR than patients with common *EGFR*-mutations, but incomplete PFS/OS data in this small cohort rendered difficult the comparison [24]. In this regard, our patient with exon 19-insertion showed no OR to erlotinib, but one cannot exclude that the concomitant *TP53*-mutation and possibly the low-level *MET*-amplification associated with MET-overexpression influenced this lack of response.

We also addressed whether possible co-mutations in alternative cancer-drivers could interfere with the response to erlotinib. In a previous exploratory study of 197 consecutive NSCLC cases with TKI-sensitive *EGFR*-mutations, 11 exhibited primary resistance to EGFR-TKIs, but apart from three of these cases exhibiting either concomitant p.T790M, *MET*-amplification, or *ALK*-fusion, no other co-mutations in driver genes that could explain the intrinsic TKI-resistance were identified by targeted NGS [25]. Of the 21 erlotinib-treated NSCLCs that we assessed by targeted NGS, 71% exhibited concomitant mutations in alternative cancer-drivers before TKI-therapy. The most frequent were *TP53*-mutations found in 67% of these cases, whereas 60% of them harbored co-mutations in either *MET*, *KRAS*, *SMAD4*, *PIK3CA*, *CTNNB1*, *NRAS, DDR2, ERBB4*, *FGFR1*, or *FGFR3*. Using the same targeted NGS platform as ours, two other groups recently detected cases of advanced *EGFR*-mutant NSCLC that prior to gefitinib-treatment displayed co-mutations very similar to those identified in our study and at comparable frequency. Notably, these cases responded significantly worse to gefitinib than those without co-mutations [26, 27].

Moreover, in a recent large database-study assessing characteristics and outcomes of patients harboring multiple molecular alterations, patients with *EGFR/KRAS* and *EGFR/PIK3CA* co-mutations experienced worse PFS during TKI-therapy than patients having only *EGFR*-mutations [6]. Collectively, these results indicate that the concurrent mutations we have detected are among the most frequent in advanced therapy-naïve *EGFR*-mutant NSCLC and could be involved in primary resistance to gefitinib and erlotinib.

Mutant *TP53* occurs in over 50% of pulmonary ADCs [8] and may interfere with TKI-induced cell-cycle arrest and apoptosis, thereby contributing to acquired TKI-resistance development [28–31]. Regarding intrinsic TKI-resistance, though, relatively small cohorts of gefitinib- or erlotinib-treated NSCLC-patients comparable to ours have shown only a marginal negative impact of coexisting *TP53*-mutations on the OR to TKIs [26, 27]. Similarly, we found no significant association between *TP53*-mutations and sensitivity to erlotinib. These findings may be due to stochastic variations related to relatively few observations and/or the type of identified *TP53*-mutations that may differently interfere with the effect of TKIs.

As for *MET*-status in the *EGFR*-mutated NSCLCs, we found 1 case with *MET*-mutation, four cases with different *MET*-amplification levels and MET-overexpression, and one case with MET-overexpression not associated with amplification. The overall frequency (22.2%) of *MET* GCN gain in our cohort of *EGFR*-mutated cases was similar to that reported by Schildhaus et al. in their overall NSCLC population and subgroup of *EGFR*-mutated tumors [11]. Also consistent with Schildhaus et al. [11], we found almost complete concordance between *MET*-amplification and MET-overexpression (4/5 cases). Furthermore, *TP53*-mutation occurred in 3/5 of patients with *MET*-amplification and/or MET-overexpression, suggesting possible growth advantages for NSCLCs that concomitantly have altered EGFR-, MET- and p53-dependent signaling. In preclinical models, *MET*-amplification has been shown to be a driver for NSCLC growth and survival [32]. In the clinical setting 5-20% of NSCLC patients with acquired resistance to EGFR-TKIs have *MET*-amplification likely due to clonal selection by TKI-treatment of preexisting *MET*-amplified cells, resulting in activated MET-signaling, which bypasses EGFR-blockade and induces cell proliferation and survival [3, 4, 33]. Although this role of *MET* in acquired TKI-resistance is well established, the potential impact on the intrinsic TKI-resistance is less clear [3, 4]. Single cases of NSCLC presenting with concomitant *EGFR*-mutation and *de novo MET*-amplification associated with primary resistance to erlotinib have been reported. Interestingly, these cases have shown response to a dual EGFR/MET blockade mediated by erlotinib/crizotinib combination [34, 35], thereby illustrating feasibility and therapeutic potential of combinatorial strategies in *EGFR*-mutant NSCLC with activation of bypass signaling pathways, such as *MET*-amplification. However, we found no indication in our cohort that *MET*-status at baseline inevitably hampered the efficacy of erlotinib-treatment. Only the case with *EGFR* exon 19-duplication (p.I744_K745insKIPVAI) that concomitantly harbored a *TP53*-mutation (p.T155P; classified as “neutral” in COSMIC) and low-level *MET*-amplification together with MET-overexpression, did not respond to erlotinib (Tables 1 and 2). The other four cases with *MET*-amplification and/or MET-overexpression, two of which also had *TP53*-mutations, as well as the case with *MET*-mutation, all partially responded to erlotinib (Tables 1 and 2). Although concurrent *EGFR*-mutation and *ALK*-rearrangement in lung ADC is more frequent than initially anticipated [7–10, 36], we detected no ALK fusion-protein expression, *i.e*. no sign of *ALK*-rearrangement, in any of the 23 *EGFR*-mutated patients. Consistent with the reported possibility of rare concomitance of *EGFR*- and *KRAS*-mutations in lung ADCs [7, 8, 12, 13], one of our *EGFR*-mutated cases harbored the p.G12V *KRAS*-mutant together with the p.R158L *TP53*-mutant, and somehow surprisingly it partially responded to erlotinib. *KRAS*-mutations are one of the most common genetic events in lung ADC and constitutively activate effectors downstream of EGFR, thus they can potentially cause TKI-resistance and be a negative predictive biomarker for response to EGFR-TKIs in NSCLC [37]. Nevertheless, the role of *KRAS*-mutations in intrinsic TKI-resistance remains unclear. Another of our cases partially responding to erlotinib displayed *TP53*- and *PIK3CA*-mutations concurrently with the activating p.L858R EGFR-mutant. Somatic mutations in the catalytic domain of *PIK3CA* have been implicated in acquired TKI-resistance and also found in 1%–3% of NSCLCs prior to TKI therapy [38]. These *PIK3CA*-mutations render *EGFR*-mutant NSCLC cell lines resistant to EGFR-TKIs by activating AKT-signaling and inhibiting TKI-induced apoptosis [38]. In a large, retrospectively analyzed cohort of patients with advanced *EGFR*-mutant lung ADC, the concomitant occurrence of a *PIK3CA*-mutation was a negative prognostic factor associated with decreased median OS, but did not impact benefit from EGFR-TKI monotherapy in terms of objective RR, PFS and duration of response [39]. As our erlotinib-treated *PIK3CA*-comutated case, these data suggest that *PIK3CA*-mutations despite being considered cancer-drivers do not necessarily represent a mechanism of primary resistance to erlotinib in *EGFR*-mutated NSCLC.

Two other patients partially responding to erlotinib displayed co-mutation of the *CTNNB1* gene coding for β-catenin, the main effector in the Wnt/β-catenin signaling pathway that transactivates cell proliferation-related genes [40]. Interestingly, preclinical data show that β-catenin is activated by *EGFR*-mutants and contributes to the development of *EGFR*-mutated NSCLC, so that *CTNNB1*-mutations can potentially induce resistance to EGFR-TKIs [41–43]. Therefore, targeting the Wnt/β-catenin pathway might provide novel strategies to counteract TKI-resistance [41]. However, the role of *CTNNB1*-mutations in TKI-resistance awaits further clinical confirmation.

Partial response to erlotinib was also seen in a case concomitantly revealing an *EGFR* exon 19-microdeletion, “pathogenic” mutations in *TP53* and *ERBB4* and “unknown”/unreported mutations in *NRAS* and *DDR2* (patient 15, Table 2). *NRAS*-mutations are detectable in ~1% of NSCLCs, particularly ADCs in current/former smokers [44], but their role in TKI-resistance, despite being hypothetically similar to that of *KRAS*-mutations, is poorly explored. Missense mutations of *DDR2*, which encodes the collagen receptor discoidin domain receptor 2, are present in 4% of pulmonary SCCs and approximately 1.5% of ADCs (http://cancer.sanger.ac.uk/cosmic). However, their frequency was reported increased to 16% in *EGFR*-mutated NSCLC, without significant impact on gefitinib-treatment [26]. *ERBB4*-mutations occur in ~1% of NSCLCs (http://cancer.sanger.ac.uk/cosmic). The observed p.S239P mutation resides in ERBB4 extracellular dimerizing domain, has formerly been reported in esophageal cancer as ERBB4-activating mutation [45] and might represent a bypass-mechanism for erlotinib-resistance, but the role of *ERBB4*-mutants in TKI-resistance needs further clarification. Finally, three of the 23 *EGFR*-mutant NSCLCs harbored a concomitant mutation in either *SMAD4*, *FGFR1*, or *FGFR3*. The former encodes the SMAD4 transcriptional co-factor that mediates TGF-β tumor-suppressive function by inducing growth arrest and apoptosis [46], but the incidence and function of inactivating *SMAD4*-mutations in TKI-resistance are scantly known. Recently *SMAD4* co-mutations were observed in *EGFR*-mutated NSCLCs especially occurring in gefitinib-sensitive patients [26, 27]. However, our patient with *SMAD4* co-mutation exhibited mixed response to erlotinib. Thus, the significance of *SMAD4*-mutations in TKI-resistance remains unclear.

Constitutive FGFR1-activation by *FGFR1*-amplification, -translocation or -mutation is associated with various malignancies. *FGFR1*-amplification has been observed in up to 20% of pulmonary SCCs and less frequently in ADCs and small-cell carcinomas [47]. Single preclinical and clinical studies suggest that activated FGFR1-signaling may represent a mechanism of acquired resistance to EGFR-TKIs [48–50]. Lim et al. reported that 2 of 20 *EGFR*-mutant NSCLCs not responding to gefitinib carried a concomitant *FGFR1*-mutation [26]. Our *FGR1*-mutated case concurrently displayed an intronic substitution in *TP53* predicted to be “pathogenic” in COSMIC and nonetheless did show OR to TKI-treatment.

Activating *FGR3*-mutations have been reported in small fractions of advanced pulmonary SCCs and ADCs [51–53], including cases with co-existing *EGFR*-mutations. Additionally, lung ADC may contain oncogenic *FGFR3-TACC3* fusions that may function as bypass-mechanism associated with intrinsic/acquired resistance to EGFR-TKIs reversible by FGFR-inhibitors [54–56]. As we recently described elsewhere, our advanced *EGFR*-mutant ADC-case with concomitant *FGFR3*-mutation displayed mixed response, with pleural metastasis progressing already 7 weeks after initiating first-line erlotinib-treatment, whereas other metastatic sites progressed only 6 months later after acquiring additional p.T790M *EGFR*-mutation [57].

Somehow surprisingly, among our 23 *EGFR*-mutated patients treated with erlotinib, 17 showed OR (complete/partial) regardless of the presence of cancer-relevant co-mutations that can potentially represent mechanisms of primary resistance to EGFR-TKIs, because they occurred in genes coding for proteins that either are directly downstream the EGFR or belong to alternative by-pass pathways, as discussed above. No statistically significant difference in PFS or OS was observed when stratifying according to concomitant driver status, potentially due to the low number of patients included (Figure 2). Although concurrent aberrations in other driver genes did not seem to significantly impair erlotinib efficacy in most of our cases, they could potentially decrease the time to progression by serving as alternative driver-pathways. Once treated with erlotinib, NSCLCs may become resistant either by selecting pre-existing subclones carrying resistance-mutations such as p.T790M or possessing the ability to depend on alternative oncogenic pathways for survival [3, 58]. Thus, acquired TKI-resistance may already exist in subclones at baseline and targeting a single activating driver mutation will eventually lead to treatment failure, while combining different pathway-targeted drugs based on pre-treatment molecular analyses could potentially be applied in NSCLC to avert or postpone the appearance of resistant tumor cells. In this regard, our study presents limitations, such as the fairly low number of investigated *EGFR*-mutated cases and the fact that the utilized techniques (NGS, FISH, IHC) covered only a specified number of driver genes. In contrast, the optimal implementation of combination targeted therapy for NSCLC in the future will also require broader knowledge of other genetic or epigenetic events that potentially can represent additional targets or resistance mechanisms and thereby serve as predictive biomarkers as well. This will obviously add to the complexity of combinatorial therapeutic strategies, nevertheless, implementing broad genomic sequencing panels, such as the recently reported MSK-IMPACT assay, can detect multiple potentially actionable targets coexisting within individual tumors in a significant amount of patients, further underlining the future need of combination therapies against NSCLC [5].

In conclusion, the vast majority of advanced *EGFR*-mutated NSCLCs in our cohort showed co-mutations in other cancer driver genes before receiving first-line erlotinib-treatment. These concurrent alternative mutations may not necessarily lead to initial resistance to erlotinib but they may impact the time to progression to first-line TKI-treatment and represent potential therapeutic targets for combined targeted therapy.

## MATERIALS AND METHODS

### Tumor samples and mutation analysis

Five hundred and fourteen consecutive NSCLCs of all stages diagnosed at our institution from July 2013 to August 2015 were included. All tumors of non-squamous cell type were examined, while squamous cell carcinomas (SCCs) were only tested on specific clinical indication (patients younger than 50 years or never smokers/light smokers with less than 2 pack-years). DNA was extracted from two 5-μm-thick formalin-fixed paraffin-embedded (FFPE) tissue sections of diagnostic biopsies or from smears of cytological samples obtained from the primary tumor or metastases in lymph nodes or distant organs. Samples diagnosed as ADCs and judged suitable (minimal relative tumor cell nuclei content of 20%) were routinely tested for *EGFR*-mutations using the Cobas® EGFR Mutation Test (Roche Diagnostics) on the Cobas z480 analyzer (analytical sensitivity of 5%) according to the supplier´s instructions. This fully automated analysis covers 41 *EGFR*-mutations, including G719X (G719A, G719C, or G719S) in exon 18, 29 variable exon 19-microdeletions, S768I and T790M in exon 20, five different exon 20-insertions, and L858R in exon 21 (two variants).

Specimens below the 20% cut-off were enriched for tumor content using manual micro-dissection.

Samples from *EGFR*-mutated NSCLC patients that were candidate for first-line TKI treatment (advanced stage, PS 0-2) were further tested by next generation sequencing (NGS) for other possible simultaneous cancer-relevant gene mutations and for confirming and specifying the type of *EGFR*-mutations detected by Cobas® Test, whereas cases not eligible to TKI therapy (low stage/operable or receiving chemo-radiotherapy, PS 3, or deceased before therapy start) were not further analyzed. For each FFPE specimen, 10 ng of genomic DNA, purified by the QIAamp DNA Minikit (Qiagen) and quantified by the Qubit® dsDNA HS assay on a Qubit® 2.0 Flourometer (Thermo Fisher Scientific), were used for library preparation with the Ion AmpliSeq Library Kit 2.0 (LifeTechnologies) and the Ion AmpliSeq Colon-Lung Cancer Research Panel v2 (Ion Torrent, Thermo Fisher Scientific). This panel yields 92 amplicons covering 504 mutational hot-spot regions and 1825 hot-spot mutations in the following 22 lung/colon cancer-associated genes: *AKT1* (NCBI reference sequence: NM_005163), *ALK* (NM_004304), *BRAF* (NM_004333), *CTNNB1* (NM_001904), *DDR2* (NM_001014796), *EGFR* (NM_005228), *ERBB2* (NM_004448), *ERBB4* (NM_005235), *FBXW7* (NM_033632), *FGFR1* (NM_023110), *FGFR2* (NM_022970), *FGFR3* (NM_000142)*, KRAS* (NM_033360)*, MAP2K1* (NM_002755)*, MET* (NM_001127500)*, NOTCH1* (NM_017617)*, NRAS* (NM_002524)*, PIK3CA* (NM_006218)*, PTEN* (NM_000314)*, SMAD4* (NM_005359)*, STK11* (NM_000455) and *TP53* (NM_000546). Library concentration was determined by the Ion Ampliseq™ TaqMan Quantification Kit.

Preparation of sequencing templates and Ion Spheres followed by loading on Ion 316 chips v2 was automatically performed on the Ion Chef System (Thermo Fisher Scientific). Sequencing was carried out on an Ion Torrent Personal Genome™ (PGM) sequencer using the Ion PGM Sequencing 200 Kit v2 according to the manufacturer's instructions. Data analysis, including alignment to the hg19 human reference genome and variant calling, was performed by the Torrent Suite Software v.4.4 (Thermo Fisher Scientific) and visually verified with the Integrative Genomics Viewer; IGV v.2.1 (Broad Institute)

### Fluorescence *in-situ* hybridization (FISH) and immunohistochemistry (IHC)

FISH was utilized to detect possible *MET*-amplification, which can represent a mechanism of TKI-resistance [59].

Two-μm-thick FFPE tissue sections from biopsies or cell-blocks of cytological samples were hybridized overnight with the Zyto-Light SPEC MET/CEN7 Dual Color Probe (ZytoVision) that detects the *MET*-gene and the centromeric portion of the *MET*-harboring chromosome 7, as previously described with minor modifications [11].

Briefly, slides were scanned using a X63 objective and appropriate filter sets (DM5500 fluorescent microscope; Leica), using normal fibroblasts, leukocytes, endothelial cells or non-neoplastic lung tissue as internal controls and individually analyzing 100 tumor cell nuclei (20 neighboring tumor cell nuclei from five random areas of homogenous distribution of *MET* signals) with the X100 objective counting *MET* (green) and CEN7 (orange) signals. FISH was assessed by one reader (ES-R) without knowing the IHC data. The tumor samples were classified into the following four groups of *MET*-amplification status [11].

A) *High-level amplification* when they displayed a *MET*/CEN7 ratio ≥2.0 or an average *MET* gene copy number (GCN) per cell of ≥6.0 or ≥10% of tumor cells containing ≥15 *MET* signals. B) *Intermediate-level of GCN gain* when ≥50% of cells contained ≥5 *MET* signals and the criteria for high-level amplification were not fulfilled. C) *Low-level of GCN gain* when ≥40% of tumor cells showed ≥4 *MET* signals and criteria for high-/intermediate-level amplification were not fulfilled. D) *Negative* (*no amplification/GCN gain*) when none of the above criteria were fulfilled.

IHC for membranous and cytoplasmic expression of MET-receptor was performed on FFPE 2.5-μm-thick tissue sections using a BenchMark ULTRA automated slide immunostainer (Ventana Medical Systems Inc.; item no. N750-BMKU-FS), Ultra Cell Conditioning solution (CC1) pretreatment (Ventana) for 8 min at 95°C, four CC1 treatments (20, 36, 52, and 64 min), and incubation with the pre-diluted CONFIRM anti-Total c-MET (SP44) Rabbit mAb (Ventana Medical Systems, Inc.; Cat. # 790-4430) for 16 min, as described [11]. The reaction was visualized using ultraView DAB Detection Kit and hematoxylin counterstaining (Ventana). MET expression was scored in a blinded manner (without knowing the FISH results) by one observer (ES-R), assessing staining intensity (negative, weak, moderate, or strong) and the percentage of cells with these intensities using bronchial/alveolar epithelial cells as internal controls for weak-moderate intensity. Thereby, 4 diagnostic “immunoscores” were defined: 3+/overexpression (strong intensity in ≥50% of tumor cells); 2+ (moderate intensity in ≥50% of tumor cells); 1+ (weak intensity in ≥50% of tumor cells); and 0 (no staining or <50% of tumor cells stained with any intensity) [11].

For ALK immunostaining, as indicator of *ALK*-rearrangement [60], the fully automated IHC assay using the pre-diluted VENTANA anti-ALK (D5F3) Rabbit mAb (Ventana Medical Systems Inc.; Cat. # 790-4794) was used together with the Optiview DAB IHC detection kit and Optiview Amplification kit on the Benchmark XT stainer (Ventana Medical Systems Inc.), implementing a binary scoring system (present/absent moderate/strong granular cytoplasmic staining in any percentage of tumor cells) according to manufacturer's instructions [11, 60].

Each case was also stained with an unrelated matched rabbit IgG mAb used as negative control. Samples with known *MET*-amplification and MET-overexpression or *ALK*--rearrangement and ALK-overexpression were used as positive controls [61].

### Assessment of tumor response and definition of lack of response/intrinsic resistance

Objective response (OR) evaluation was done by CT-scan at baseline (maximum 1 month prior to TKI-treatment start), followed by CT-scans every 9 weeks. RECIST v1.1 criteria for definitions of response and progression were used [62]. Patients who progressed within 18 weeks (4 months) were classified as having intrinsic resistance.

### Statistical analysis

Overall survival (OS) was defined as the time from onset of treatment to the time of death from any cause or last follow-up. PFS was defined as the time from onset of treatment with erlotinib to a documented progression or death from any cause. For patients without any progression at the time of analysis, the date of last follow-up was considered right-censored. Kaplan-Meier curves were used to estimate curves for OS and PFS and chi-square test was used for comparison of proportions. A p-value < 0.05 was considered statistically significant.

The study was approved by the institutions' local ethical committee and was with data collection on pre-defined case report forms.
